# Immunity1Read before the Nebraska Veterinary Medical Association.

**Published:** 1898-08

**Authors:** A. T. Peters

**Affiliations:** Lincoln, Nebraska


### THE JOURNAL

OF

### COMPARATIVE MEDICINE AND VETERINARY ARCHIVES.

VOL. XIX. *AUGUST, 1898.* No. 8.

### IMMUNITY.[1]

BY A. T. PETERS, D.V.M.,
LINCOLN, NEBRASKA.

THE title of my paper is "Immunity." It is a subject that has attracted a great deal of attention in the medical world, both abroad and in this country, since the time of that master of vaccination, Jenner, and that pioneer, Pasteur, who really demonstrated the power of immunity when he showed his results of vaccination against anthrax in 1881. Since then vaccination has been practised in various diseases with marked success. But it has been found that immunity may be produced not only by vaccination, but also by the sera-fluids, and this subject of serum-therapy has been uppermost in the minds of medical men since the discovery of Behring's diphtheritic and tetanus antitoxin. Both have been greatly modified in the technique of preparation, and there have been theories and theories advanced as to their relation to immunity. So I will endeavor to discuss immunity produced not only by vaccination, but also by the sera-fluids.

Immunity may be natural, hereditary, or acquired. It would be useless for me to go into minute details in regard to natural and inherited immunity, so we will give the most of our attention to acquired immunity, as that concerns us in combating contagious diseases.

*Inherited Immunity.* As an example of inherited immunity, I will refer you to the Algerian sheep. All who are acquainted with the literature of anthrax know that this sheep is immune from anthrax, and can be pastured on infected land that is extremely fatal to other species. This breed, we find, inherit their immunity.

[1] Read before the Nebraska Veterinary Medical Association.

*Natural Immunity.* To illustrate natural immunity I will repeat what experimenters in the laboratory have for a long time known, that field-mice are not susceptible to septicæmia. Another instance that is familiar to all veterinarians is that no case is recorded in the history of veterinary medicine in which the cow has contracted glanders.

*Acquired Immunity.* Acquired immunity may be effected by the recovery from an attack of a disease or by inoculation against subsequent attacks. But acquired immunity is not always effected in this way, as certain diseases are recurrent. For instance, one attack of malaria renders a person even more susceptible to a second attack. Consequently, inoculations in such cases cannot produce immunity. On the other hand, it has been clearly demonstrated in New York, and also in our own laboratory, that diphtheria convalescents carry the diphtheria-bacilli in the sputa or excreta with impunity. It has also been reported that cholera patients have passed the coma-bacillus for weeks after recovery.

In inoculating against diseases there are many things to influence susceptibility and immunity. Age appears to exert some influence, as young animals are more susceptible than older ones. This is especially true in the case of hog-cholera. For the last four years our records show that over 88 per cent. of the hogs that succumbed weighed from 50 to 150 pounds. This is also true with horses and influenza.

The strength of the virus is also an important factor. It is well, therefore, to know the strength of your virus and dose accurately, according to the size of the animal. For example, it would not do to inoculate first with the second vaccine of anthrax and omit the first, for you would certainly meet with disaster. The temperature also plays an important part. For instance, the normal temperature of the fowl is 107.3°. Consequently, the fowl is exempt from anthrax until the body-temperature is reduced by artificial means so that the germ can grow.

Heredity likewise exerts considerable influence in certain cases in the production of immunity. Duclert found in sheep-pox that animals found an immune mother possessed either relative or absolute immunity. To show the effect of an immune father on the offspring, Ehrlich made six experiments with an immune father and susceptible mother, with the result that not even a temporary immunity was transferred to the young. But even the mother does not always transmit immunity. We have made experiments along this same line. At conventions of swine-breeders the question is often raised as to whether or not a litter of pigs would be susceptible to hog-cholera if the sow had had the cholera at the time of pregnancy. There seemed to be a difference of opinion among the stockmen, so it was decided by our department to buy a litter of pigs that were from a sow that went through an attack of the cholera at the time of pregnancy, and place them in infected herds where undoubted cholera existed. This was done, and in every case the pigs died. From this experiment, together with the statements of stockmen who have since observed the same, I think that the progeny of a mother immune against hog-cholera are not immune, nor do I think that they are in any way rendered less susceptible to the disease. In some diseases, as smallpox and measles, one attack gives the subject an immunity that may last him a lifetime. On the other hand, an attack of diphtheria or relapsing fever gives an acquired immunity that lasts only for a limited period, after which there is danger of a second attack. In still another type of disease no immunity whatever is produced. These diseases are tuberculosis, leprosy, and cancerous diseases, and although vaccination has been tried it was of no avail. Hospital patients treated with tuberculin seem to recover for a period of one or two years, after which time the disease breaks out with renewed vigor. So, to assure acquired immunity by vaccination, we must use cultures of those microbes which produce a large amount of toxin, and not those which produce no symptoms of poisoning when the pathological microbe is present.

*Theories as to the Cause of Immunity.* The duration and cause of immunity depend on factors of which we have no certain knowledge. The different investigators have expounded different theories. The most noted is the hypothesis of Metchnikoff's famous theory of phagocytosis. He says that the cells of the body, such as the phagocytes, have the power of destroying by digestion the microörganisms that invade the circulation, and thus protect the body from infection. If, on the other hand, the phagocytes are incapable of preventing the growth of the infectious organisms, the bacteria get the upper hand and the animal suffers fatal infection. Again, if the amœboid cells succeed in warding off the attack, they habituate themselves to the action of the germs and are thus able to resist this germ as long as the immunity lasts, the time varying, as has been shown, in different diseases.

Büchner has put some restrictions on this theory since he has shown that after the leucocytes are destroyed by freezing the bactericidal power of the blood still remains. He later concludes that although leucocytes do furnish a germicidal power they do not do so as a rule by actual digestion of the germ. Other observers have since noted that the bactericidal power of the blood is contained in the leucocytes.

Pasteur explained the phenomenon in this way: The pathological microörganism of each specific disease finds a special pabulum on which to live, and so soon as this becomes used up it can no longer exist. Hence the animal remained immune until this pabulum was renewed in the system.

Chauveau explained the matter from a chemical standpoint. He suggested that acquired immunity was due to a waste-product of the germ, which remained in the system, and in which the germ itself could not live. This is illustrated by the yeast-plant, which in growing produces alcohol, and which, in turn, prohibits further growth of the germ.

All these theories have been vigorously attacked by Behring, who has done so much in serum-therapy in the last seven years. His theory, which has been quite generally accepted, is that the host elaborates a specific antitoxic substance which chemically neutralizes or antagonizes specific toxins as acids neutralize bases.

The latest theory at our command is the so-called "granular theory," that there are present in the blood free granules derived from the leucocytes that destroy the pathogenic germ. This has just been recently advanced in a paper read before the Johns Hopkins Hospital Medical Society, October 18, 1897, by Drs. Stokes and Wegefarth. They conclude by advancing the following theory: "The bactericidal power of the leucocytes of the blood, and of the serum of man and many animals, is due to the presence of specific granules, especially the eosinophilic and neutrophilic. When called upon to resist the action of invading bacteria the granular leucocytes can give up their granules to the surrounding fluids or tissues. Not only does this enable us to understand how apparently cell-free fluids can destroy bacteria, but the production of the alexin by the leucocytes also affords a better explanation of the hyperleucocytosis of infection so strongly urged by Metchnikoff, and by no means disproves the supposition that the leucocytes can take up bacteria either while alive or after being destroyed by means of the germicidal granules."

But no matter how much these theories differ and antagonize each other, they all agree in one thing—that the germicidal power of the blood lies somewhere in the serum. In accordance with this fact, the investigations and discoveries of Behring have shown that if the toxin of a pathological organism be injected in proper quantities for a sufficient length of time into the body of one of the higher animals, the blood-serum of this animal acquires specific antitoxic properties; that is, a given quantity of this serum injected into another susceptible animal would render it immune. And it is by this means that the medical profession is scoring one of the greatest triumphs in modern medicine.

I will now endeavor to discuss the more important diseases in which immunity may be acquired by vaccination or by the introduction of blood-serum from immune animals.

*Rabies.* Pasteur's treatment of rabies is undoubtedly known to all of you. His cure consisted in killing rabbits suffering from rabies and injecting an emulsion of the old and desiccated cord into the system of the animal he wished to immunize. This first injection was followed by repeated injections of fresher and fresher cords until absolutely fresh and virulent cords could be injected with no effect. Since the time of this discovery thousands of patients have been treated by this method, and institutes for this purpose have sprung up all over the world.

*Anthrax.* Pasteur found that by exposing the anthrax-bacillus to abnormal heat it gradually lost its virulence. Furthermore, he noted that sheep inoculated first with a bacillus of little virulence, and secondly with one of greater virulence, were protected against subsequent inoculation of a virulent culture that proved fatal in an animal not so protected. A public test of this experiment was made, in which twenty-five sheep were vaccinated by this method; the other twenty-five were left unvaccinated, and all were publicly inoculated with a virulent culture of the anthrax-bacillus. The result was a perfect success. All the unvaccinated animals died of anthrax, the vaccinated ones were not even ill. Since 1881 vaccination for anthrax has been practised all over the world, and "in France alone, during the twelve years succeeding its introduction, the saving has been estimated at about 300,000 sheep and over 20,000 head of cattle."

*Black-leg.* Black-leg was for a long time thought to be identical with anthrax, but through the labors of Arloing, Cornevin, and Thomas, a specific germ for this disease has been isolated. I do not think it wise to take up too much time in the discussion of the efficiency of vaccination in this disease; but I would like to emphasize the fact that all veterinarians should thoroughly acquaint themselves with the method, what has been done here and abroad toward decreasing the annual loss, which is greater than is generally thought. If the veterinarian will do this, and compare the results with those obtained by using the old remedies, he will stick to vaccination and become a useful man in the community where black-leg exists.

*Pleuro-pneumonia.* Vaccination is practised in pleuro-pneumonia in Europe, where the disease is known to exist. I quote from an article by Ecrivain in the *Veterinary Journal*, vol. xlii., No. 251: "It has not yet been fully determined how long the immunity lasts, but still, if it carries over the outbreak it would save the life of many a healthy animal. The virus is taken from the interlobular connective tissue of the lung in the first stage of a mild attack, when it will be loaded with a semifluid exudate. The fluid is collected, filtered, and put into sterilized tubes which are afterward sealed by the blowpipe. The tip of the tail is the part selected, and scarification is quite sufficient on the under surface, one drop of the filtrate being quite sufficient for protection. There has been some argument whether inoculation produces the same disease or not, but whatever it produces it is a great satisfaction to know that it produces immunity."

*Variola.* Variola runs a mild course in cattle and renders the animal immune. Persons milking cows with this disease may become infected through an abrasion, and thus become immune against a subsequent attack of this disease, as well as against human smallpox.

*Tetanus.* The serum-treatment of tetanus has interested the veterinary profession for the last seven years. It was hoped for a time that the antitoxic serum would be effective as a curative agent, but the recent experiments and data at hand have clearly demonstrated that when the symptoms of clonic spasms have set in the serum has only a limited curative effect. Professor Nocard states that it is a good prophylactic. It should be used, therefore, in localities where tetanus is known to exist, in cases where valuable horses received deep-seated punctures that have a tendency to heal on the outside, as the bacillus is anaërobic, so that wounds with good drainage are not so liable to infection.

*Rinderpest.* Germany is especially fortunate in having strict sanitary regulation in combating rinderpest, but this still runs unchecked to a great extent in Africa, Russia, and certain parts of Austria. Recently the English Government has secured the services of Dr. Koch to investigate this disease in Africa. The report of his work is more or less familiar to all of you, who have, no doubt, watched it with unbounding interest. At first the treatment consisted in the injection of serum from animals that had recovered, but those animals that received the serum showed only a limited protective power. So the next step was to try vaccination. This was done as follows : The bile was taken from an animal immediately after death, so that no putrefying germs were present, and placed in a sterilized flask and treated with glycerin. Animals treated with this experimentally could, after certain days, receive virulent blood under the skin with impunity, while the control-animals died. It has been shown that the protective power lasts three months in cases where glycerinated bile was injected. But even if the immunity were absolute only for this short period, it would be practical to inoculate the animals every three months if the disease is suspected in the neighborhood. The glycerinated bile is used because the disease cannot be spread by its use, and, secondly, great economy is effected by the use of glycerin, and, thirdly, the mixture will keep. Pure bile is undoubtedly the most powerful, but even that is not so powerful in producing immunity as the disease itself, from which immunity lasts in Russia about five years. The French experts claim that by using 100 c.c. of serum to a dose an immunity lasting two months will result.

Drs. Turner and Kolle, who have continued the experiments begun by Dr. Koch, have experimented with serum and with defibrinated blood, the action of both being found precisely similar. They conclude that infection must be assured of as definite and of as severe a character as possible, and at the same time administer the serum in such a dose as to insure the safety of the animal. This is done by injecting 1 c.c. of virulent blood on one side of the animal, and immediately after 5, 10, 20 c.c. of the serum on the other side. The animal suffers from a modified form of the disease, showing all the usual symptoms, and is salted.

*Texas fever.* The recent investigations in Texas fever in this country and in Australia are all fresh in your memories, as the cause of this disease has only recently been agreed upon. Dr. F. L. Kilborne deserves a great deal of credit, as he pointed out in 1889 and 1890 that the tick was the cause of the spread of the disease. It took some time before the stockmen and scientists would believe that the innocent-looking tick was at the bottom of it all. Many of you will recall men saying, "No tick, no Texas fever," and you will also recall what a warm reception was tendered the gentleman who made such a remark. But, however fishy it sounded then, I think the work of the Missouri Experiment

Station has now confirmed the tick-theory" beyond the shadow of a doubt. I mention this disease in connection with immunity, because Dr. Connaway, of the Missouri Station, has performed experiments in which serum was taken from naturally immune Southern cattle and injected into susceptible Northern cattle with the view of producing immunity. One animal was immunized in this way and withstood the disease when infected with the tick. The various experiments made along this line do not as yet give sufficient evidence to justify one in stating that the serum will prove an absolute prevention, but the reports are encouraging, to say the least, so that we may hope for a great revelation in the cattle-industry of the South.

*Hog-cholera.* Probably the disease most interesting to stockmen is hog-cholera. Vaccination against this disease has been in vogue for some years, but recently experiments have been made with the serum-treatment. Dr. Lorenz, of Darmstadt, Germany, has experimented with serum taken from immune rabbits and pigs, which proved successful, and his method was bought by the Government of Prussia for the purpose of making their own serum and establishing stations for that purpose all over the country. These experiments have been watched with marked interest by all who are familiar with the foreign literature. It has been recently announced that he has secured a chemical from the serum which, he claims, contains the protective power. Dr. Paul Toepper, B. F. W., No. 44, 97, announces that he has inoculated animals with this protective chemical and has not had very good success with it.

The results of experiments with serum from animals refractory to the disease, carried on at the Nebraska Experiment Station in 1896, were reported before this body last year. These experiments have been continued through 1897, about 2000 doses having been sent out. At the present time I cannot state the exact number treated and the results of the same, as the reports are not yet at hand, but the reports we have received so far are very encouraging over the previous year's results. We have given a virulent culture in connection with the serum, and have demonstrated that by this method we could not give the disease so that the slightest symptoms were shown. We have also treated piggy sows by this method without producing abortion or any deleterious effects. By this combination of the toxin and the serum we have succeeded in lengthening the period of immunity, which accounts for the better results.

In our study of the efficiency of serum we have in the past year learned many new things, one of the most important of which is the fact concerning filtered serum. Our laboratory and field experiments showed that serum lost some of its protective power by filtering it through a porcelain bougie. We have also shown, to our own satisfaction, at least, that serum alone has a much more limited power of immunity than has serum in combination with virulent blood.

We have made only a few experiments with dried serum, and they were laboratory experiments on rabbits. But those that have used the dried diphtheritic serum claim that it is unreliable. The principal objection to it from a laboratory standpoint is that it is not readily soluble in water, and this fact alone, I think, would condemn its universal use.

We are watching with interest the experiments of a German investigator, who is trying to make a more concentrated serum, and thus lessen the size of the dose. The idea is to get rid of the water that the serum contains by some means that will not injure the protective properties of the material. He has shown that by freezing the serum a darker portion settles to the bottom of the flask. This it is found is the protective part, while the watery portion remains as a layer on top. We have observed this in our own laboratory, and have also noticed that some serum is very difficult to freeze, hence we conclude that it contains less water and is possibly a stronger serum.

I have tried to demonstrate to you the value of acquired immunity, and bring out some of the latest investigations that seem to speak so highly for the sera-fluids. I am ready to answer any questions that I can, and thank you for your attention.

